# CRISPR-Cas12a assay for rapid and specific detection of *Shigella flexneri 2a* in clinical samples

**DOI:** 10.1128/jcm.01615-25

**Published:** 2026-07-20

**Authors:** Jipei Liao, Yun Su, Feng Jiang

**Affiliations:** 1 Department of Pathology, University of Maryland School of Medicine 12264, Baltimore, Maryland, USA; 2 Department of Medicine, Nanjing University of Chinese Medicine 66478, Nanjing, China; 3 BIOTARGET DX LLC, Baltimore, Maryland, USA; University of Western Australia, Perth, Australia

**Keywords:** CRISPR-Cas12a, Shigella, rapid detection, point-of-care diagnostics, RPA

## Abstract

*Shigella flexneri 2a* is the most common cause of shigellosis, a major public health concern in developing countries. Rapid and reliable diagnostic tools are critical for timely outbreak detection and management. Leveraging clustered regularly interspaced short palindromic repeats (CRISPR) technology, we developed a CRISPR-Cas12a-based assay for the rapid and specific detection of *S. flexneri 2a* and validated its performance using stool specimens from patients. Two guide RNAs targeting the *gtrII* and *gtrX* genes, unique markers of the *S. flexneri 2a* serotype, were designed to ensure specificity. Recombinase polymerase amplification (RPA) was coupled with Cas12a-mediated collateral cleavage for signal amplification, with detection by fluorescence or lateral flow. Analytical sensitivity, specificity, and clinical accuracy were compared with conventional PCR using purified DNA and 588 clinical stool specimens. The CRISPR-Cas12a assay achieved a detection limit of 10 copies/µL, comparable to PCR, and showed 100% analytical specificity without cross-reactivity to other bacteria. The isothermal reaction operated at room temperature and was completed within 1 h. Both readouts allowed visual interpretation without specialized equipment. Clinical validation of the CRISPR-Cas12a assay demonstrated a diagnostic sensitivity of 95% and specificity of 98%, comparable to PCR when evaluated using the same clinical specimens. This study provides two key advances: it establishes a CRISPR-Cas12a assay specifically targeting *S. flexneri* 2a, the predominant serotype, and validates it using a large clinical cohort. The assay’s simplicity, speed, and high diagnostic accuracy make it a valuable tool for clinical diagnostics and field-based surveillance in resource-limited settings.

**IMPORTANCE:**

Rapid and accessible diagnostics are essential for effective management of infectious diseases such as shigellosis. We developed a clustered regularly interspaced short palindromic repeats (CRISPR)-Cas12a-based assay that specifically detects *Shigella flexneri 2a*, the predominant serotype responsible for the global disease burden. This assay integrates isothermal amplification with CRISPR-mediated detection to achieve low-copy detection (10 copies/µL) within 1 h, eliminating the need for complex instrumentation. Dual fluorescence and lateral-flow readouts enable flexible use in both clinical laboratories and low-resource settings. The method’s simplicity, accuracy, and adaptability demonstrate the practical potential of CRISPR diagnostics for point-of-care applications. By enabling rapid, on-site identification of *S. flexneri 2a*, this approach can significantly improve clinical diagnosis and strengthen public health responses to enteric pathogen outbreaks.

## INTRODUCTION

Shigellosis, an acute bloody diarrhea caused by the Gram-negative, entero-invasive bacterium *Shigella* spp., poses a significant public health challenge, particularly in developing countries. Each year, Shigella infections result in approximately 60,000 deaths among children in these regions, contributing to both acute and chronic health issues (1). The burden of *Shigella* is compounded by the rise in antimicrobial-resistant strains, which poses a challenge to current treatment strategies (1). The most prevalent strain is *Shigella flexneri*, particularly serotype *2a* (*S. flexneri 2a*), which accounts for approximately 30%–40% of *S. flexneri* infections and about one-third of all shigellosis cases in low- and middle-income countries (2). The predominance of this strain necessitates the development of effective detection methods and vaccines (3).

Current methods for detecting *S. flexneri 2a* include culture-based, serological, and PCR techniques (4). Culture-based methods, though considered the gold standard, often require several days to yield results and necessitate well-equipped laboratories and skilled personnel, which are not always available in low-resource areas (4). Serological methods, which detect antibodies or antigens related to *S. flexneri 2a*, can provide faster results but often suffer from potential false positives or false negatives. PCR-based methods offer high sensitivity and specificity and can deliver results more quickly than culture-based methods; however, they require sophisticated equipment, stable electricity, and trained laboratory technicians, limiting their widespread use in developing countries. Therefore, it is crucial to develop faster and reliable tests for *S. flexneri 2a*, which can be effective in resource-limited settings to enable timely intervention and reduce disease spread.

Clustered regularly interspaced short palindromic repeats (CRISPRs) are segments of prokaryotic DNA that feature repetitive sequences (5–7). These sequences, along with CRISPR-associated proteins (Cas), form an immune system capable of targeting and cleaving specific nucleic acids, a mechanism now utilized in gene editing (5–7). Previous studies reveal that Cas proteins can also degrade DNA and RNA, allowing ultra-sensitive nucleic acid detection (5–7). Techniques, such as DETECTR and SHERLOCK, developed using Cas12a and Cas13a, have shown promise in detecting viruses like HPV, ZIKV, and DENV (5, 6). Our research demonstrates that CRISPR-Cas12a can detect a broad spectrum of viruses and genomic mutations with sensitivity comparable to that of PCR (8–12). Additionally, we developed a microplate-based CRISPR assay for simultaneous detection of multiple targets (8–13). Moreover, CRISPR has shown potential in detecting bacterial pathogens (14, 15). Cas12a-based assays can detect *Shigella flexneri* in food or artificially spiked samples; however, these methods target *S. flexneri* at the species level rather than specifically detecting *S. flexneri 2a*. Furthermore, their validation using human clinical specimens remains limited (16–18). In this study, we developed and evaluated a CRISPR-Cas12a-based assay for the rapid and specific detection of *S. flexneri 2a*. Compared with previous studies, our work provides two major advances: it establishes a CRISPR-Cas12a assay specifically targeting *S. flexneri 2a*, the predominant serotype, and validates its performance using human stool specimens, thereby demonstrating its clinical applicability in both healthcare and resource-limited settings.

## MATERIALS AND METHODS

### Bacterial strains

Nucleic acid preparations of bacterial strains, including S. *flexneri, S. boydii, S. sonnei*, and *S. dysenteriae*, as well as the non-Shigella strain *Escherichia coli* (Table 1), were obtained from the American Type Culture Collection (ATCC; Bethesda, MD). Genomic DNA isolated from the human melanoma cell line MDA-MB-435 (ATCC) was used as a negative control. The bacterial DNA was quantified and subjected to a 10-fold serial dilution in the negative control DNA, ranging from 10,000 copies/μL to 0.1 copies/μL.

**TABLE 1:** Bacterial strains

Organism	ATCC number	Serotype
*S. boydii*	9207	1
*S. sonnei*	25931	1
*S. dysenteriae*	13313	1
*S. flexneri*	700930	*2a*
*S. flexneri*	12022	*2b*
*E. coli*	NCTC 900111	–^*a*^

^*a*^ –, not applicable.

### Recombinase polymerase amplification and lateral flow readout of CRISPR-Cas12a activity for detection of *S. flexneri*

**Fig 1:**
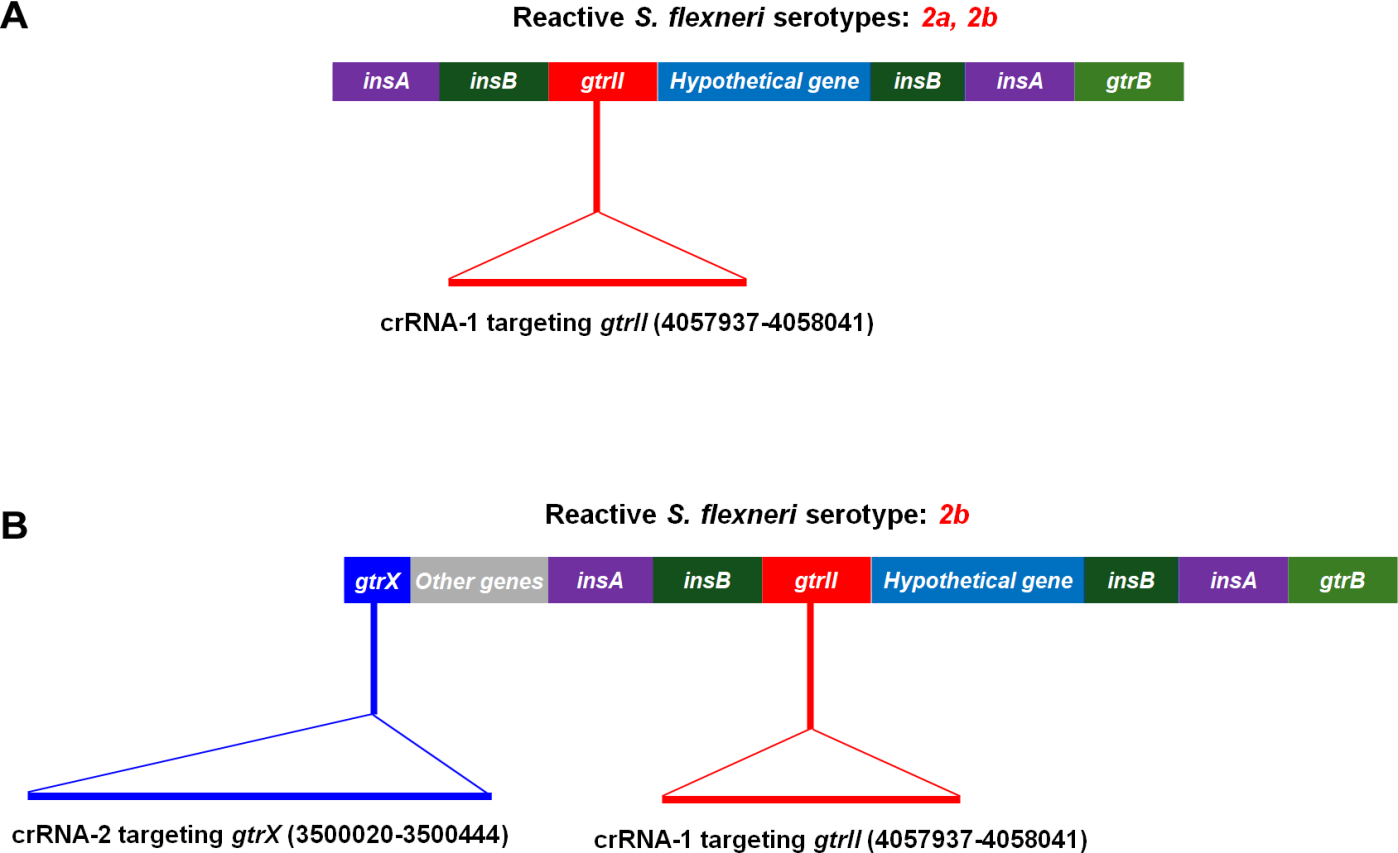
Gene Alignment and CRISPR target sites in *Shigella flexneri* serotypes. (**A**) The gene alignment in *S. flexneri 2a and 2b* and the location of crRNA-1 to specifically target the *gtrII* gene. The crRNA target sites were selected based on the presence of a canonical LbCas12a TTTV PAM sequence. The genes are labeled as follows: *insA*, *insB, gtrII*, *Hypothetical gene*, and *gtrB*. The crRNA-1 target region within the *gtrII* gene (base pairs 4057937–4058041) is highlighted, indicating the specific target site for crRNA-1 in the CRISPR assay. (**B**) The gene alignment in *S. flexneri 2b* and the locations of crRNA-1 and crRNA-2 to specifically target the *gtrII* and *gtrX* genes, respectively. The genes are labeled as follows: *tnpA*, *gtrB*, *gtrA, gtrX*, *gtrII*, a*rcA,* and *int*. The crRNA-2 target region within the gtrX gene (base pairs 3500020–3500444) and the crRNA-1 target region within the *gtrII* gene (base pairs 4057937–4058041) are highlighted, indicating the specific target sites for crRNA-1 and crRNA-2 in the CRISPR assay.

We performed CRISPR experiments to detect bacteria by modifying our protocols as described in previous studies (7–12). As shown in Table 1, there are numerous serotypes of *S. flexneri*, which share highly similar or identical genomic regions. In the serotypes, the *gtrII* gene exists in both *S. flexneri 2a* and *S. flexneri 2b*, while the *gtrX* gene exists in *S. flexneri 2b, 1d, 3a, 5b*, and *X*, but not in *S. flexneri 2a*. To develop a CRISPR assay for the specific detection of *S. flexneri 2a*, we designed primers for recombinase polymerase amplification (RPA) to specifically amplify the target DNA segments of *gtrII* and *gtrX,* respectively (Table 2). To design crRNAs that can specifically identify the sequences within the two genes (Table 2) (Fig. 1), multiple candidate crRNAs targeting the *gtrII* and *gtrX* genes were designed and evaluated *in silico* based on sequence conservation, predicted specificity, and the presence of a canonical LbCas12a protospacer-adjacent motif (PAM; TTTV) (Table 3). Candidate target sites were also aligned to *S. flexneri* reference genomes to minimize potential cross-reactivity with other Shigella species or serotypes. The crRNAs were then experimentally screened using purified genomic DNA templates, and those exhibiting optimal signal intensity and specificity were selected for further validation.

**TABLE 2:** Genes commonly used for identification of various *Shigella flexneri* serotypes*^a^*

Genes	Accession no.	Reactive *S. flexneri* serotype(s)
*** gtrII***	AY900451	***2a, 2b***
*** gtrX***	L05001	*1d,* ***2b*** *, 3a, 5b, X*
* gtrV*	CP000266	*5a, 5b*
* wzx6*	EU294165	*6*
* oac*	X59553	*1b, 3a, 3b, 4b, 5a, 5b, 7b*
* gtrI*	AF139596	*1a, 1b, 1d, 7a, 7b*
* gtrIc*	FJ905303	*7a, 7b*
* gtrIV*	AF288197	*4a, 4b*
* ipaH*	M76445	All Shigella species and serotypes

^*a*^ Italicized bold text shows the genes to be targeted in the CRISPR analysis for the detection of *S. flexneri 2a* and *2b*.

**TABLE 3:** Primers for RPA and crRNAs for *S. flexneri* detection

Component	Target gene	Sequence (5' → 3')	Type
Forward-1	*gtrII* (*2a*/*2b*)	CAAACGACTCAGGAAATATGC	RPA primer
Reverse-1	*gtrII* (*2a*/*2b*)	AATTCATAAATGCAACCATCCT	RPA primer
crRNA-1	*gtrII* (*2a*/*2b*)	UAAUUUCUACUAAGUGUAGAU-CAAAAGUGUCUGCGCUCAUG	Full crRNA
crRNA-1-1	*gtrII* (*2a*/*2b*)	UAAUUUCUACUAAGUGUAGAU-CCCACUCCAUGAGCGCAGACA	Full crRNA
crRNA-1-2	*gtrII* (*2a*/*2b*)	UAAUUUCUACUAAGUGUAGAU-AGGAGAAAGCAGUGAGUGGAU	Full crRNA
Forward-2	*gtrX* (*2b*)	AATGCTGGATGGGATAATCACCTT	RPA primer
Reverse-2	*gtrX* (*2b*)	GAGACGGCTTCTCCATGTTTTGCT	RPA primer
crRNA-2	*gtrX* (*2b*)	UAAUUUCUACUAAGUGUAGAU-CGGUUGCAAGUGUUGGCAGU	Full crRNA
crRNA-2-1	*gtrX* (*2b*)	UAAUUUCUACUAAGUGUAGAU-CCGCAAUAUCGCCCUGCAAC	Full crRNA
crRNA-2-2	*gtrX* (*2b*)	UAAUUUCUACUAAGUGUAGAU-GCAGGUAUUCAGGGUUAGUCC	Full crRNA

RPA was performed by mixing 5 µL of DNA with 0.48 µM forward and reverse primers, 29.5 µL primer-free rehydration buffer, 5 µL DNA from boiled liquid samples, and 14 mM magnesium acetate (Sigma-Aldrich, St. Louis, MO) in a 50-µL reaction using the TwistAmp Basic kit (TwistDx, Cambridge, UK). Preliminary time-course experiments were conducted to evaluate RPA incubation duration. The RPA incubation temperature (37°C) was used based on manufacturer recommendations and our prior experience with CRISPR-based detection assays, as described in published studies (8–12). Incubation beyond 20 min did not yield a measurable increase in signal intensity but was associated with increased nonspecific background. Therefore, a 10–20 min incubation window was selected to balance assay sensitivity, specificity, and turnaround time in this study. For the CRISPR reaction, 5 µL of the RPA reaction was incubated with 100 nM LbCas12a (Integrated DNA Technologies [IDT], Coralville, IA) and 100 nM crRNAs at 37°C, then diluted in binding buffer along with a dual-labeled ssDNA substrate tagged with FAM and biotin (IDT). The reaction mixture was combined with 80 µL of HybriDetect buffer (TwistDx), and a Milena HybriDetect1 lateral flow dipstick (TwistDx) was inserted into this mixture. Since Cas12a cleavage and signal generation occurred only in reactions containing successfully amplified target DNA, reactions lacking RPA amplification did not produce detectable lateral-flow signals. Results were observed with the naked eye and captured using a smartphone camera. A positive result was indicated by a significant signal increase on the test line (upper line) and a decrease on the control line (lower line). Negative controls included no-template controls (NTC) and non-target bacterial genomic DNA controls (*Escherichia coli*). These controls were included in every assay run to monitor nonspecific amplification and Cas12a-mediated background activity. A separate non-targeting crRNA was not used, as the combination of no-template and non-target DNA controls provided a stringent assessment of assay specificity under the experimental conditions used.

### Detection of *S. flexneri 2a* by CRISPR-Cas12a using a fluorescence plate reader and a UV light illuminator

Following RPA amplification, CRISPR-Cas12a detection was performed as described above using an ssDNA fluorophore-quencher reporter (IDT) instead of the FAM/biotin-labeled substrate. Identical reaction mixtures were analyzed by two methods: (i) quantitative fluorescence measurement using a BioTek Synergy H1 plate reader and (ii) qualitative fluorescence visualization under UV illumination using a Bio-Rad ChemiDoc MP Imaging System (Bio-Rad Laboratories, Hercules, CA). For visual detection, fluorescence was excited under UV light, and signal intensity corresponding to target DNA presence was assessed by eye or captured photographically. As described above, detectable Cas12a cleavage occurred only following successful RPA amplification of the target sequence, with no fluorescence signal observed in the absence of amplification. Fluorescence kinetics were analyzed for representative samples to demonstrate signal separation and assay performance.

### Contamination control measures

To minimize the risk of cross-contamination during RPA amplification and CRISPR-Cas12a detection, sample preparation, RPA setup, and CRISPR reaction assembly were performed in physically separated work areas using dedicated equipment. Aerosol-resistant filter tips were used throughout, and reagent preparation was conducted separately from sample handling. Each assay run included NTC and non-target bacterial DNA controls. Under these conditions, no evidence of carryover contamination or false-positive signals was observed across independent experiments.

### Quantitative PCR detection and data analysis of *S. flexneri 2a*

YBR Green PCR assays were performed according to a previous study with modifications (19). Briefly, SYBR Green-based quantitative PCR (qPCR) (50 µL) was performed using 1× PCR Master Mix, 5 µL of DNA, and gtrII/ipaH primers on a Bio-Rad CFX96 system. Cycling conditions were 95°C for 10 min; 40–45 cycles of 95°C for 10 s, 54.7°C for 10 s, and 60°C for 60 s; followed by 72°C for 10 min. Melt-curve analysis confirmed specificity, and Ct ≤35 was considered positive. To specifically identify *S. flexneri 2a*, a two-gene analytical strategy was applied following the method of Liu et al. (19). The ipaH gene served as a genus-level marker for all Shigella species, while the gtrII gene identified isolates belonging to serogroup 2 (*S. flexneri 2a* or *2b*). A second PCR targeting the gtrX gene was used to differentiate between the two serotypes: samples positive for gtrII but negative for gtrX were classified as *S. flexneri 2a*, whereas samples positive for both gtrII and gtrX were classified as *S. flexneri 2b*. The primer sequences and diagnostic purposes are listed in Table S1.

### Study population and specimens

A total of 588 stool specimens (385 *S. flexneri 2a*-positive and 203 *Shigella*-negative) were analyzed. Positive specimens were obtained from patients presenting with acute diarrhea (<96 h duration, with mucus and ≥10 WBCs per high-power field) at Jiangsu Province Hospital of Chinese Medicine. All procedures were conducted under institutional review board-approved protocols, following the ethical standards. The demographic and clinical characteristics of the study population are summarized in Table S2. The mean ± SD age of patients was 3.5 ± 1.0 years, while that of controls was 3.6 ± 0.9 years. The male-to-female ratio was approximately 1:1 in each group. All participants had no history of antibiotic exposure prior to sample collection. Infection with *Shigella flexneri 2a* was confirmed using standard bacteriological methods, including culture on MacConkey and XLD agar, followed by serological identification (serotyping), as previously described (19, 20). Negative specimens included culture-negative and *ipaH/gtrII*-negative stool samples from age- and sex-matched diarrheal patients or healthy volunteers.

### Validation of CRISPR-Cas12a assay for detection of *S. flexneri 2a* in stool specimens

**Fig 2:**
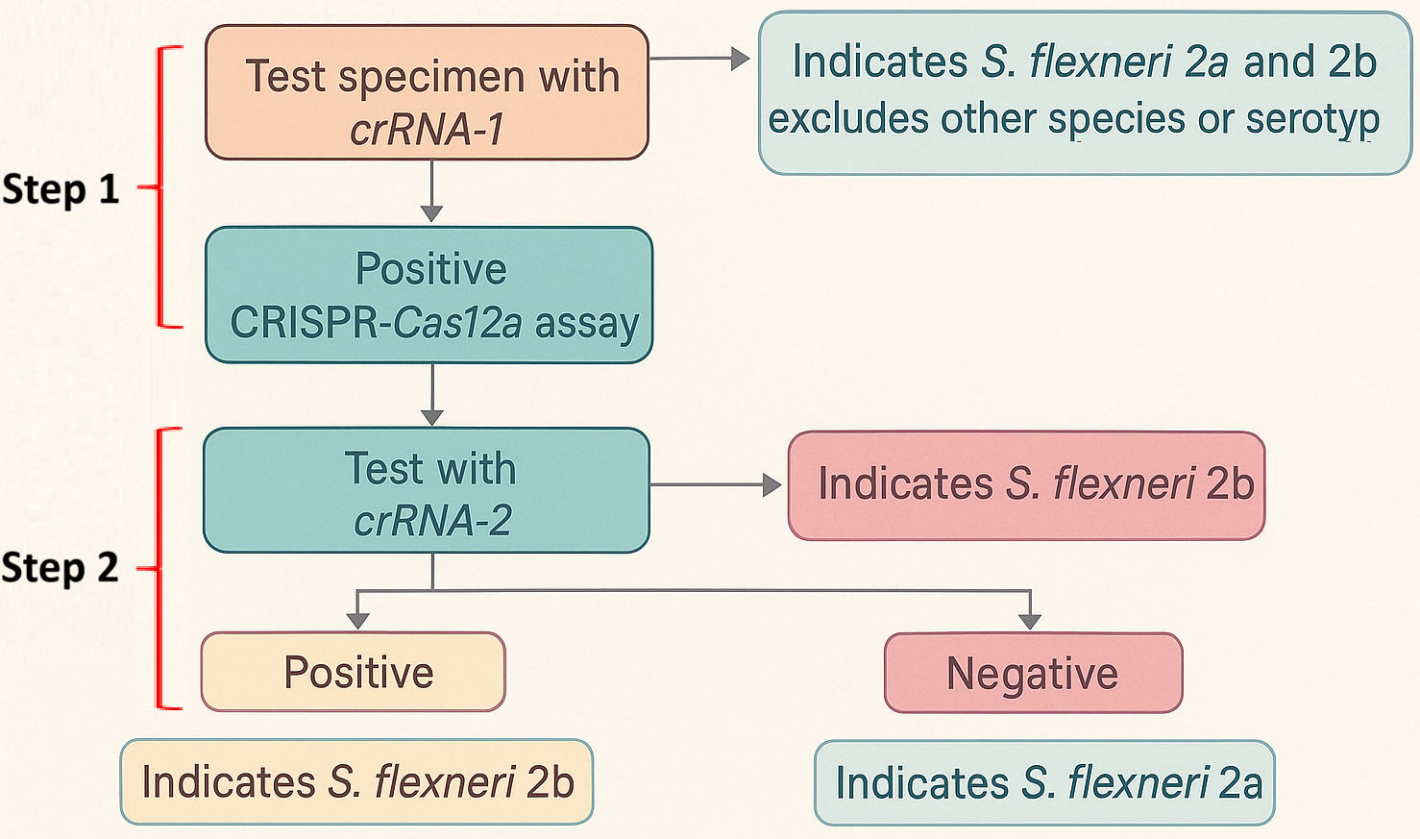
Workflow of the two-step CRISPR-Cas12a assay for specific detection of *S. flexneri 2a*. Step 1: a screening assay using crRNA-1 targeting the *gtrII* gene. A positive signal indicates the presence of *S. flexneri* serotype 2 (*2a* or *2b*). Samples that are negative in Step 1 are interpreted as *Shigella*-negative and are not tested further. Step 2: a confirmatory assay applied only to Step 1-positive samples, using crRNA-2 targeting the *gtrX* gene. A positive result confirms *S. flexneri 2b*, whereas a negative result confirms *S. flexneri 2a*. Cas12a activation requires successful RPA amplification of the target sequence; therefore, application of Step 2 in the absence of Step 1 amplification does not generate a false-positive signal. Because Step 2 detection depends on amplification of the *gtrX* gene, Step 2-positive results cannot occur in samples that are negative in Step 1.

To process stool samples for nucleic acid extraction, approximately 200 mg of stool was homogenized in 1 mL of phosphate-buffered saline (Thermo Fisher, Waltham, MA) and centrifuged at 12,000 × *g* for 2 min. The supernatant was heated at 95°C for 5 min to inactivate pathogens (21). DNA was extracted using the QIAamp Fast DNA Stool Mini Kit (Qiagen, Germany) according to a previous report (22), and eluted in 200 µL AE buffer and stored at −20°C until analysis. For clinical validation, lateral-flow strip readout was used for all specimens and served as the basis to determine diagnostic sensitivity and specificity. Fluorescence plate reader measurements and UV-based visual detection were used for qualitative confirmation of assay performance and signal concordance, but were not used independently to derive diagnostic accuracy metrics. Each reaction set included a positive control (*S. flexneri 2a* ATCC 700930 DNA), a negative control (*E. coli* NCTC 9001 DNA), and NTC. The assay was implemented using a predefined sequential workflow (Fig. 2) in which Step 2 testing was performed exclusively on samples that yielded a positive signal in Step 1. This decision rule was applied consistently across all clinical specimens, and all samples were also tested by qPCR using the established protocol of Liu et al. (19).

### Statistical analysis

The analytical performance of the CRISPR-Cas12a assay was evaluated using serially diluted *S. flexneri 2a* DNA (10⁴−0.1 copies/μL) to determine the limit of detection and by testing multiple Shigella species and *E. coli* strains to assess analytical specificity. Fluorescence intensity values were expressed as mean ± standard deviation (SD), and differences were analyzed using two-tailed Student’s *t*-tests, with *P* <0.05 considered statistically significant. Analytical sensitivity was evaluated empirically using serial dilutions of *S. flexneri* 2a genomic DNA tested in triplicate. The limit of detection (LOD) was defined as the lowest concentration consistently detected across replicates. We determined the detection limit by direct testing in the lab rather than by using advanced statistical modeling or specialized digital PCR methods. For clinical validation, based on the anticipated assay performance (sensitivity ≥ 0.90 and specificity ≥ 0.95) and a desired precision of ±3%, approximately 385 positive and 203 negative specimens were estimated to be required to achieve >90% statistical power for diagnostic validation. Sensitivity and specificity were determined from 2×2 contingency tables, and agreement with the qPCR assay was assessed using Cohen’s κ coefficient. All analyses were performed using GraphPad Prism (version 10.0).

## RESULTS

### CRISPR-Cas12a strategy for specific detection of *S. flexneri 2a*

**Fig 3:**
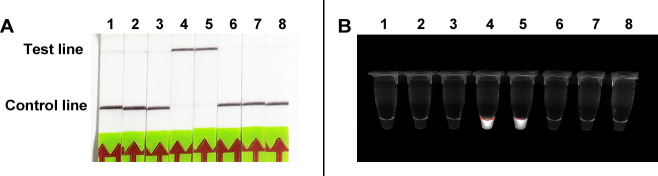
Analytical specificity of the CRISPR assay for detection of *S. flexneri* using reference bacterial genomic DNA. (**A**) The paper strips display the results for various bacterial strains. A positive result is indicated by a significant signal increase on the test line (upper line) and a decrease on the control line (lower line). There was a cross-reaction between *S. flexneri 2a* and *S. flexneri 2b* (red arrows). No positive results of crRNA-1 were observed for *S. boydii 1, S. sonnei, S. dysenteriae,* and *E. coli*. Lanes: (1) *S. boydii 1*, (2) *S. sonnei*, (3) *S. dysenteriae*, (4) *S. flexneri 2a*, (5) *S*. *flexneri 2b*, (6) *E. coli*, (7) DNA of human MDA-MB-435 cells, and (8) NTC (no template control). (**B**) The results of crRNA-1 for various bacterial strains were read using a UV light illuminator. Consistent with panel A, a positive result is indicated by a visible fluorescence signal (red arrows). A cross-reaction between *S. flexneri 2a* and *S. flexneri 2b* was found. No positive results of crRNA-1 were observed for *S. boydii 1, S. sonnei, S. dysenteriae*, and *E. coli*.

**Fig 4:**
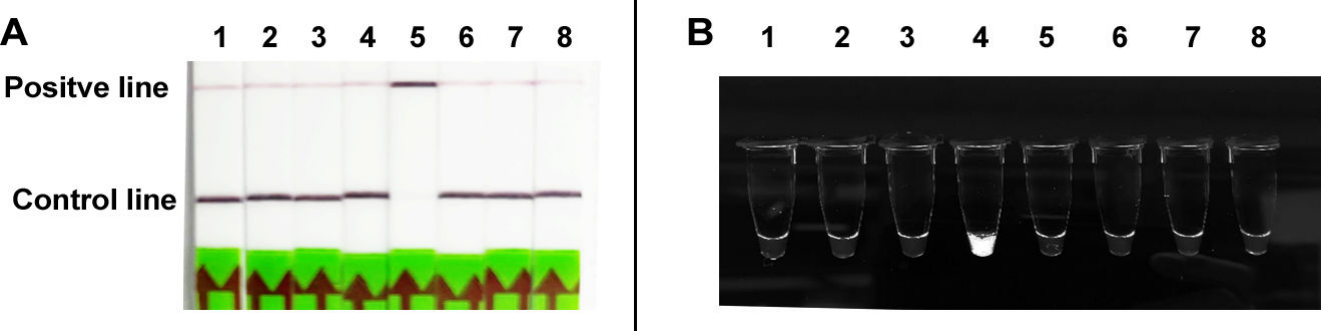
Specificity of the CRISPR assay with crRNA-2 for the detection of *S. flexneri*. (**A**) The paper strips display that only *S. flexneri 2b* is positive for crRNA-2 (red arrow), whereas *S. flexneri 2a* is negative for crRNA-2. No positive results were observed for other bacteria. Lanes: (1) *S. boydii 1*, (2) *S. sonnei*, (3) *S. dysenteriae*, (4) *S. flexneri 2a*, (5) S. *flexneri 2b*, (6) *E. coli*, (7) DNA of human MDA-MB-435 cells, and (8) NTC, no-template control. (**B**) The results of crRNA-2 for various bacterial strains are displayed using a UV light illuminator. Only *S. flexneri 2b* (lane 4) shows a positive fluorescence signal (red arrow), while *S. flexneri 2a* (lane 3) is negative for crRNA-2.

One major challenge in the molecular detection of *Shigella* is cross-reactivity caused by sequence homology in conserved regions shared with other species and among multiple *Shigella* serotypes. In *S. flexneri*, the *gtrII* gene is present in both serotypes *2a* and *2b*, whereas the *gtrX* gene is found in serotypes *2b*, *1d*, *3a*, *5b*, and *X*, but absent in *2a*. Accordingly, we designed a panel of crRNA-1s targeting *gtrII* for the detection of both serotypes *2a* and *2b*, and a panel of crRNA-2s targeting *gtrX* for specific detection of serotype *2b* (Fig. 1, Tables 2 and 3). Analytical specificity of the crRNAs was evaluated using contrived samples consisting of purified genomic DNA from multiple Shigella species and serotypes (*S. flexneri 2a* and *2b*, *S. boydii, S. sonnei, S. dysenteriae*), as well as *E. coli* as a non-Shigella control (Table 1) (Fig. 3, Fig. 4). Among the candidate crRNAs, crRNA-1 (*S. flexneri*) and crRNA-2 (*S. flexneri 2b*), demonstrating the highest analytical sensitivity and specificity, were selected for the CRISPR assay (Table 3). Quantitative fluorescence measurements demonstrated clear signal separation between *S. flexneri 2a*-positive and negative samples. Time-resolved fluorescence kinetics showed a rapid and sustained increase in fluorescence intensity in positive reactions, whereas negative and NTC reactions remained at baseline levels throughout the assay period (Fig. S1). These results confirm the robustness of Cas12a-mediated signal generation and are consistent with qualitative fluorescence and lateral-flow readouts.

Our detection strategy for *S. flexneri 2a* employs a sequential two-step CRISPR-Cas12a workflow designed to ensure both specificity and operational clarity (Fig. 2). In Step 1, samples are screened using crRNA-1 targeting the *gtrII* gene; a positive result indicates the presence of *S. flexneri* serogroup 2 (serotype *2a* or *2b*). Only Step 1-positive samples advance to Step 2, in which crRNA-2 targeting the *gtrX* gene is applied. A positive Step 2 result confirms *S. flexneri 2b*, whereas a negative Step 2 result confirms *S. flexneri 2a*. Samples negative in Step 1 are interpreted as Shigella-negative and are not subjected to Step 2 testing. This predefined sequential decision logic minimizes unnecessary reactions, reduces assay complexity, and limits the potential for cross-contamination. Furthermore, because Cas12a-mediated detection requires sequence-specific RPA amplification, Step 2 testing cannot generate a positive signal in the absence of successful Step 1 amplification, even if performed inadvertently. Together, these features define the analytical specificity of the assay using contrived samples and provide intrinsic robustness against operator error, supporting reliable implementation in routine laboratory settings.

### The CRISPR-Cas12a system can sensitively detect *S. flexneri 2a*

**Fig 5:**
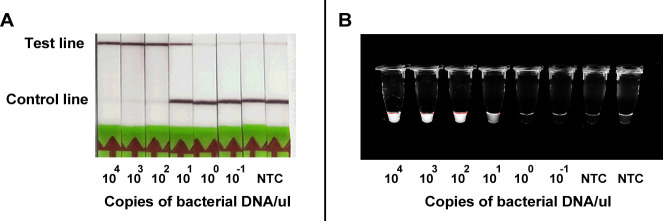
Sensitivity of the CRISPR assay for the detection of *S. flexneri*. (**A**) The test strips display the results for various concentrations of *S. flexneri* DNA from 10,000 to 0.1 copies/µL. The assay demonstrates the ability to detect as low as 10 copies/µL of *S. flexneri* DNA (red arrow). (**B**) The test tubes display the results for various concentrations of *S. flexneri* DNA, ranging from 10,000 to 0.1 copies/µL. The assay demonstrates the ability to detect as low as 10 copies/µL of *S. flexneri* DNA (red arrow). Each concentration was tested in triplicate.

To assess the analytical sensitivity of the CRISPR-Cas12a assay, genomic DNA from *S. flexneri 2a* was serially diluted 10-fold (10⁴ to 0.1 copies/µL) in a background of human genomic DNA and tested in triplicate at each concentration. The lowest concentration consistently detected across replicates was 10 copies/µL, which was defined as the analytical LOD of the assay (Fig. 5). qPCR analysis performed on the same dilution series demonstrated a comparable LOD of 10 copies/µL (Fig. 5; Fig. S2). This parallel comparison indicates that CRISPR-Cas12a has comparable sensitivity to conventional qPCR for detecting *S. flexneri*. However, the CRISPR-Cas12a assay can be performed at room temperature within 1 h, whereas PCR requires a thermocycler, different temperature cycles, and a longer reaction time of approximately 3 h.

### Diagnostic performance of the CRISPR-Cas12a assay in clinical specimens

All no-template and negative control reactions remained negative in these clinical specimens, indicating the absence of detectable cross-contamination during assay implementation. Diagnostic sensitivity and specificity values reported in Table 4 are based exclusively on lateral-flow readout results obtained from clinical stool specimens. Among the 385 culture-confirmed *Shigella flexneri 2a*-positive specimens, 365 were correctly detected by the CRISPR-Cas12a assay, whereas 20 were false negatives, yielding a sensitivity of 95% (Table 4). Of the 203 culture-negative control specimens, 199 were accurately classified as negative, with 4 false positives, resulting in a specificity of 98%. Furthermore, diagnostic performance of the CRISPR-Cas12a assay was directly compared with qPCR using the same clinical stool specimen set. The CRISPR-Cas12a assay showed sensitivity and specificity comparable to qPCR in the same specimen set (Table 4), supporting the accuracy of the CRISPR-based detection platform. In addition, lateral flow-based readouts of the CRISPR-Cas12a assay yielded distinct and easily interpretable signals within 60 min. Moreover, the CRISPR assay requires no instrumentation, operates at room temperature, needs minimal expertise, and allows immediate visual interpretation of results with the naked eye.

**TABLE 4:** Diagnostic performance metrics for both CRISPR-Cas12a and qPCR were calculated using the same set of clinical stool specimens^*a*^

Method	Parameter	*S. flexneri 2a* positive (*n* = 385)	*S. flexneri 2a* negative (*n* = 203)	Diagnostic measure (95% CI)
CRISPR-Cas12a	Positive	365 (TP)	4 (FP)	Sensitivity = 94.8% (92.1–96.8)
	Negative	20 (FN)	199 (TN)	Specificity = 98.0% (95.0–99.5)
qPCR	Positive	365 (TP)	4 (FP)	Sensitivity = 94.8% (92.1–96.8)
	Negative	20 (FN)	199 (TN)	Specificity = 98.0% (95.0–99.5)

^*a*^ TP, true positive; FP, false positive; TN, true negative; FN, false negative; CI, confidence interval. Note: diagnostic performance for CRISPR-Cas12a was determined using a lateral-flow strip readout. qPCR analysis was performed on the same clinical specimens using an established reference protocol (19).

## DISCUSSION

In this study, we demonstrate that the CRISPR-Cas12a with a pair of crRNAs can sensitively and specifically detect *S. flexneri 2a*. Furthermore, using a lateral flow dipstick or a UV light illuminator, we can rapidly visualize the results of CRISPR-Cas12a without requiring specialized technical expertise or ancillary equipment. In addition, analytical specificity was established using contrived samples of reference bacterial DNA prior to clinical validation in stool specimens. Direct comparison using the same clinical specimens demonstrated that the CRISPR-Cas12a assay achieved sensitivity and specificity comparable to those of qPCR, while offering operational advantages, such as isothermal amplification and visual readout. Moreover, the strong fluorescence and lateral-flow signals obtained within 60 min highlight the assay’s potential as a rapid, reliable, and equipment-independent diagnostic tool for detecting *S. flexneri 2a* directly from stool specimens.

Previous reports have demonstrated the feasibility of using CRISPR-Cas12a-based platforms for the detection of *S. flexneri* in food or artificially spiked samples (16–18). However, the platforms are designed to detect *S. flexneri* in general, rather than specifically targeting *S. flexneri 2a*, which represents a major serotype, accounting for up to 40% of *S. flexneri* infections (2, 18). Furthermore, validation of CRISPR-Cas12a assays using actual human clinical specimens, particularly stool samples, has remained limited. In the present study, we developed a CRISPR-Cas12a-based detection system specifically designed to identify *S. flexneri 2a* and validated its performance using clinical stool specimens from a large cohort of cases and controls. In addition, Liu et al. developed a qPCR assay for accurate identification of *S. flexneri 2a* in clinical specimens. However, despite its high analytical performance, qPCR requires expensive instrumentation, trained personnel, and time-consuming amplification and analysis steps, limiting its use in low-resource or point-of-care settings. In contrast, our CRISPR-Cas12a assay demonstrated diagnostic accuracy comparable to qPCR in clinical samples while operating at room temperature and providing results within approximately 1 h. Moreover, it enables visual detection without specialized equipment, offering a rapid, low-cost, and field-deployable alternative for effective disease surveillance and outbreak control.

Our designed crRNA-1 for identifying *S. flexneri 2a* also shows positivity for *S. flexneri 2b*. This cross-reactivity can be explained by sequence similarity, as *S. flexneri 2a* and *2b* share highly similar or identical genomic regions. However, using two distinct crRNAs in a two-step manner (Fig. 2) can efficiently distinguish between *S. flexneri 2a* and *2b*, thus providing a potential diagnostic test. To further streamline detection, we plan to apply advanced bioinformatics tools to design novel crRNAs that precisely target *S. flexneri 2a* in a simpler and more straightforward manner.

A limitation of this study is that analytical sensitivity was determined using purified *S. flexneri 2a* genomic DNA rather than stool-based spiked samples. Although this approach is commonly used to establish baseline assay performance, stool matrices can introduce inhibitory factors that affect clinical sensitivity. Accordingly, the LOD should be interpreted as an analytical LOD and not as a clinical one. Future studies will evaluate clinical LOD using stool samples spiked with defined bacterial concentrations and processed through the complete extraction and detection workflow. An additional limitation is that clinical validation focused on *S. flexneri 2a*-positive and *Shigella*-negative stool specimens and did not include clinical samples infected with other *S. flexneri* serotypes. Although analytical testing using contrived samples demonstrated clear serotype discrimination, the absence of additional serotype-positive clinical specimens limits direct assessment of clinical specificity across all *Shigella* serotypes. Future studies incorporating a broader range of clinically confirmed *Shigella* serotypes will be important to further validate serotype-level specificity in clinical matrices. Despite these limitations, the two-step CRISPR-Cas12a strategy provides a mechanistic basis for serotype discrimination that was analytically validated and may support further clinical evaluation in diverse diagnostic settings. The per-sample cost of the CRISPR-Cas12a assay is currently comparable to PCR-based molecular tests but is expected to decrease with reagent optimization, bulk production of Cas enzymes and crRNAs, and simplified assay formats. These features make the platform particularly attractive for decentralized testing environments where infrastructure, rather than reagent cost alone, is the primary limiting factor.

In conclusion, this CRISPR-Cas12a assay provides a rapid, sensitive, and equipment-independent platform for the specific detection of *Shigella flexneri 2a*. Unlike previous studies that demonstrated feasibility only in food or artificially spiked samples, this work validates the performance in actual patient-derived specimens, confirming its strong potential for point-of-care applications in the prevention and management of *Shigella* outbreaks, particularly in resource-limited clinical settings.

## Data Availability

The data that support the findings of this study are available from the corresponding author upon reasonable request.

## SUPPLEMENTAL MATERIAL

Supplemental tables and figures jcm.01615-25-s0001.pdf Tables S1 and S2, and Figures S1 and S2.

ASM does not own the copyrights to Supplemental Material that may be linked to, or accessed through, an article. The authors have granted ASM a non-exclusive, world-wide license to publish the Supplemental Material files. Please contact the corresponding author directly for reuse.
