# Supplementary material for: CRISPR-Cas12a assay for rapid and specific detection of Shigella flexneri 2a in clinical samples
Source: J Clin Microbiol. 2026 Jul 20;64(8):e01615-25. doi: 10.1128/jcm.01615-25 (PMC13464027; doi:10.1128/jcm.01615-25)
Supplement: Supplementary file 1 [file jcm.01615-25-s0001.pdf]

SUPPLEMENTAL MATERIAL

Supplementary Table 1. Primer sequences used for qPCR detection of *Shigella flexneri* 2a[19]

Target gene*	Primer name	Sequence (5′→3′)	Diagnostic role
ipaH	ipaH-F	GTTCCTTGACCGCCTTTCCGATACCGTC	Genus-level marker for *Shigella* spp. and EIEC
	ipaH-R	GCCGGTCAGCCACCCTCTGAGAGTA	
gtrII	gtrII-F	TGGATGTTGGGATGGTAGTT	Identifies serogroup 2 (*S. flexneri* 2a or 2b)
	gtrII-R	CCATCAGCATCAGTACAGCA	
gtrX	gtrX-F	CGCGAAGTTATGCGTTATGA	Differentiates *S. flexneri* 2b (+); negative result confirms *S. flexneri* 2a
	gtrX-R	CTTGTTGATGCGTGTTTGGT	

*, the *ipaH* gene was used as a pan-*Shigella* control. Detection of *gtrII* indicates serogroup 2, while absence of *gtrX* distinguishes

Supplementary Table 2. Demographic and clinical characteristics of samples

Sample characteristic	Patients (n = 385)	Controls (n = 203
Age (mean [SD]), years	3.5 (± 1.0)	3.5 (± 1.0)
Male *(%)*	193 (51.1)	103 (50.7)
Female *(%)*	192 (49.9)	100 (49.3)
Shigella-positive by standard bacteriological methods* (%)	385(100)	

*, standard bacteriological methods, including culture and serotyping; SD, standard deviation.

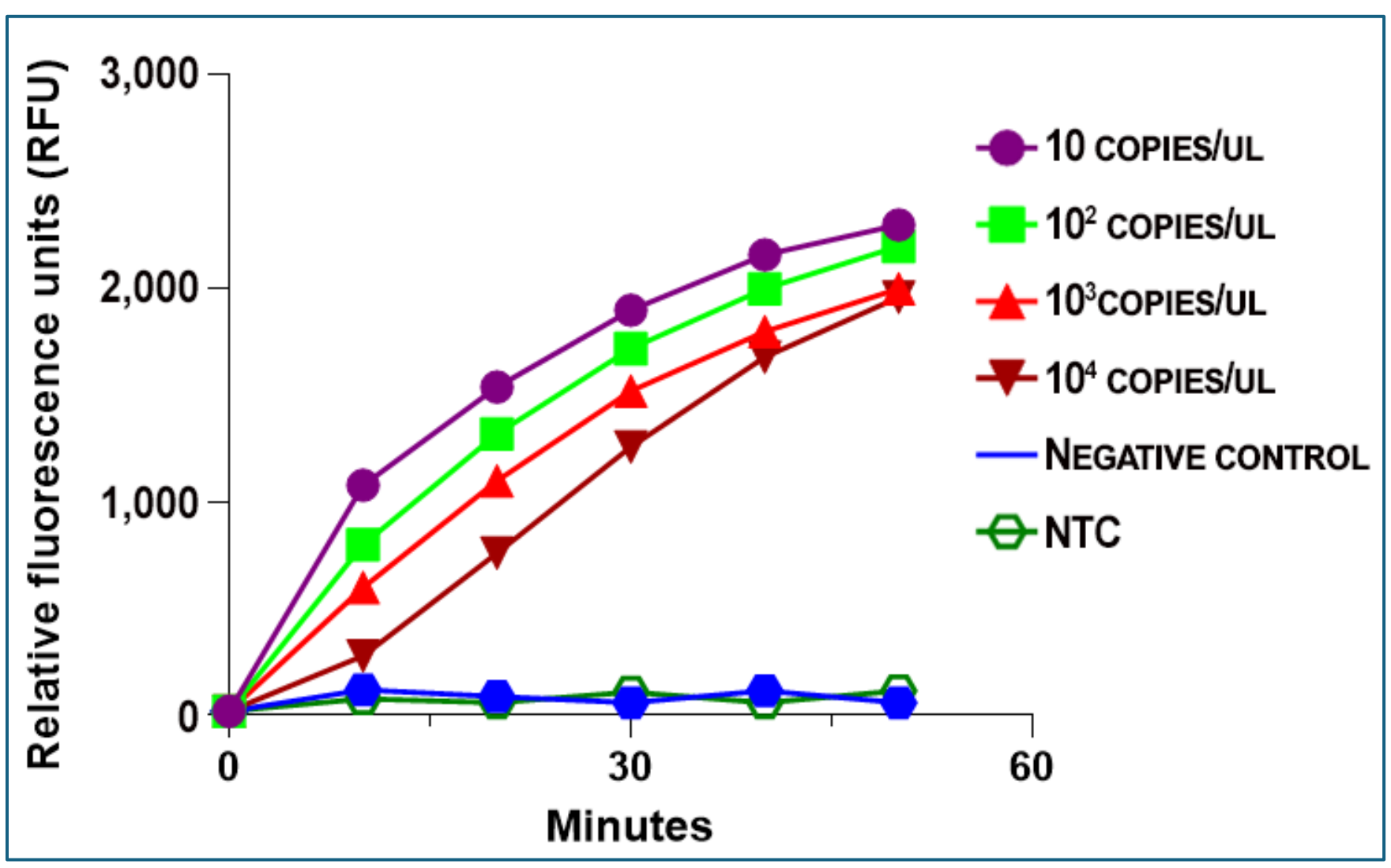

Supplementary Figure S1. Quantitative fluorescence kinetics of the CRISPR-Cas12a assay. Representative real-time fluorescence kinetics of the CRISPR-Cas12a assay for detection of Shigella flexneri. Reactions containing S. flexneri target DNA at concentrations ranging from 10^1 to 10^4 copies/μL exhibited a rapid, time-dependent increase in relative fluorescence units (RFU) over a 60-min incubation at 37 °C. Fluorescence signals from positive reactions reached similar plateau levels at later time points, consistent with reporter saturation, while remaining clearly distinguishable from negative controls. Non-target bacterial controls and no-template controls (NTC) showed baseline fluorescence with no signal amplification, demonstrating high analytical specificity.

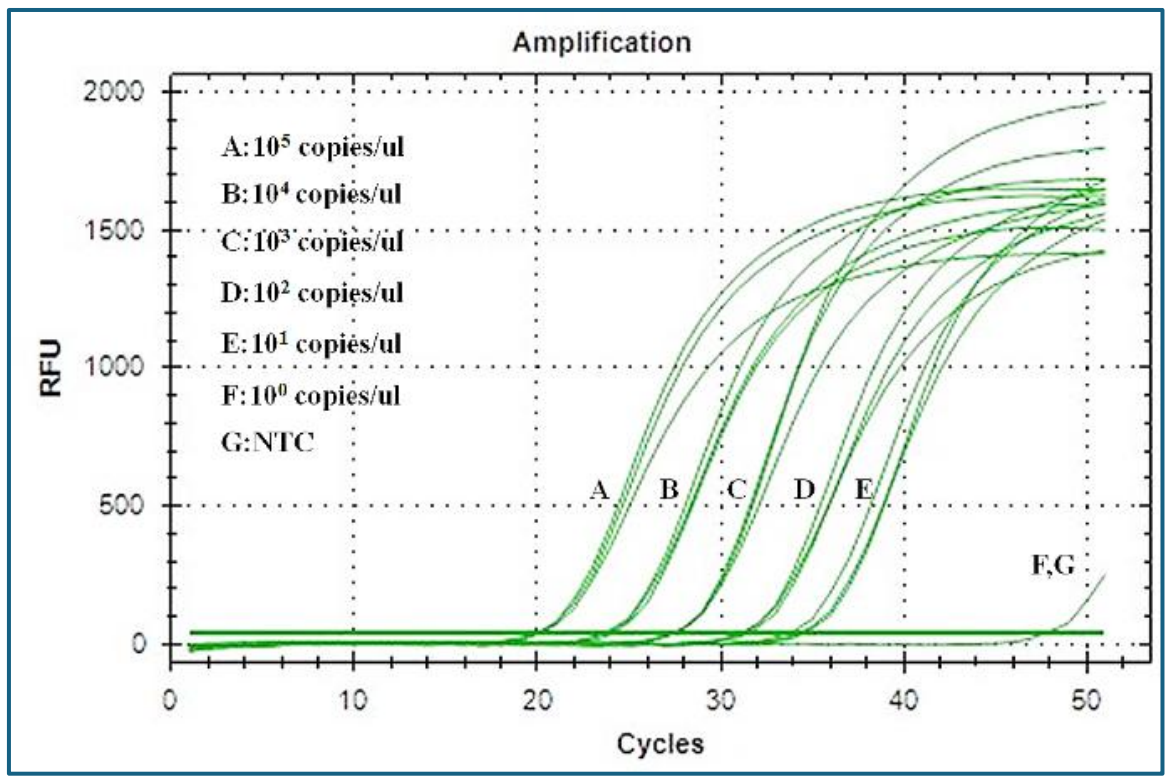

Supplementary Figure 2. PCR Amplification Curves for Different Copy Numbers of DNA of *S. flexneri.* The concentrations are labeled as follows: A: 10^5 copies/μl, B: 10^4 copies/μl, C: 10^3 copies/μl, D: 10^2 copies/μl, E: 10^1 copies/μl, F: 10^0 copies/μl, and G: NTC (No Template Control). The y-axis represents the relative fluorescence units (RFU), and the x-axis represents the number of PCR cycles. The lowest detectable copy number with a cycle threshold (Ct) of 40 is 10 copies/μl.
